# Investigating the Potential of Electroless Nickel Plating for Fabricating Ultra-Porous Metal-Based Lattice Structures Using PolyHIPE Templates

**DOI:** 10.1021/acsami.3c04637

**Published:** 2023-06-13

**Authors:** Nihan Sengokmen-Ozsoz, R. Boston, Frederik Claeyssens

**Affiliations:** † Kroto Research Institute, Department of Materials Science and Engineering, The University of Sheffield, Sheffield S3 7HQ, United Kingdom; ‡ Department of Materials Science and Engineering, The University of Sheffield, Mappin Street, Sheffield S1 3JD, United Kingdom

**Keywords:** polyHIPE, 3D printing, stereolithography, emulsion templating, electroless nickel plating

## Abstract

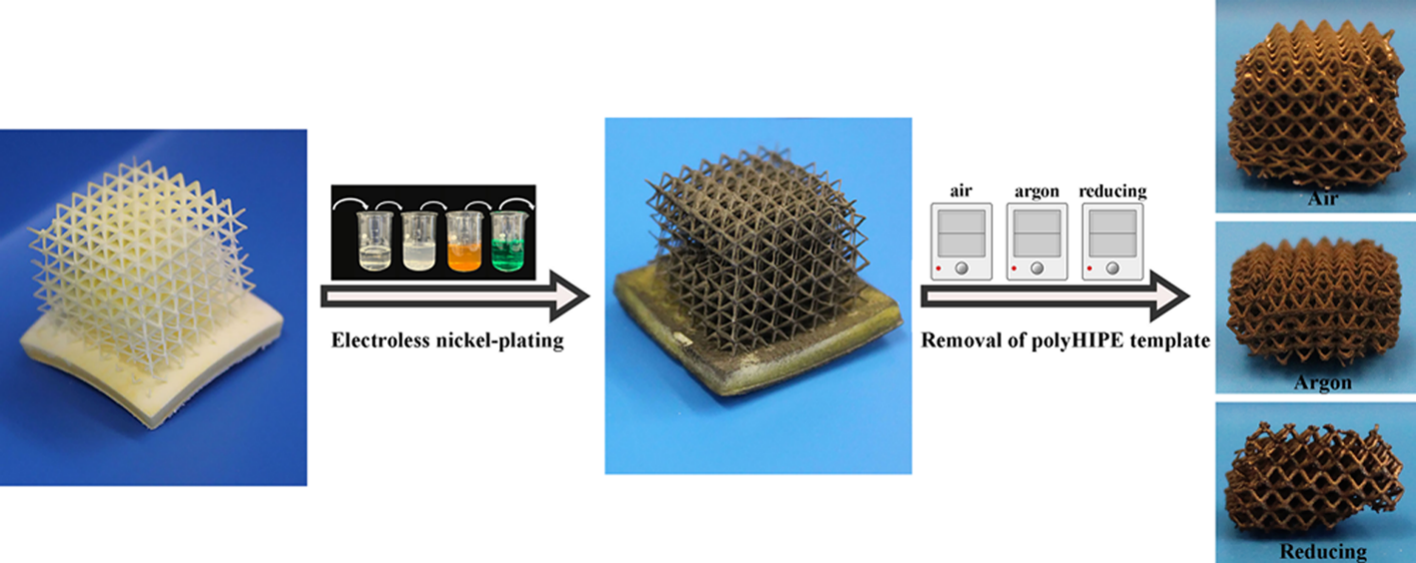

The use of polymerized high internal phase emulsions (polyHIPEs) as templates for electroless nickel plating is a promising method for producing ultra-porous metallic lattice structures with consistent wall thickness. These structures have desirable properties such as low density, high specific strength, resilience, and absorbency, making them suitable for various applications including battery electrodes, catalyst supports, and acoustic or vibration damping. This study aimed to optimize and investigate the electroless nickel plating process on polyHIPEs. Initially, a surfactant (Hypermer)-stabilized water-in-oil emulsion based on 2-ethylhexyl-acrylate and isobornyl-acrylate was used as a 3D printing resin to create polyHIPE structures. Then, the electroless nickel plating process was optimized using polyHIPE discs. The study also examined the effects of air, argon, and reducing atmospheres during the heating process to remove the polyHIPE template using metallized 3D-printed polyHIPE lattice structures. The findings indicated that different atmospheres led to the formation of distinct compounds. While nickel-coated polyHIPEs were fully oxidized in an air atmosphere, nickel phosphide (Ni_3_P) structures occurred in argon and reducing atmospheres along Ni metal. Moreover, in argon and reducing atmospheres, the porous structure of the polyHIPEs was retained as the internal structure was completely carbonized. Overall, the study demonstrated that intricate polyHIPE structures can be used as templates to create ultra-porous metal-based lattices for a wide range of applications.

## 1 Introduction

Porous structures have gained significant importance in various fields due to their unique properties, characteristics, and potential applications. On the basis of mimicking the structure of naturally occurring porous materials, various porous structures have been created, such as foams, honeycombs, and lattice structures.^1−4^

Foams, honeycombs, and lattices are cellular structures. The term “cellular structure” was commonly used to describe porous materials before the emergence of the lattice structure.^5,6^ Lattices offer numerous superior qualities, such as being lightweight, highly durable, capable of absorbing energy, dissipating heat, and minimizing vibration, aspects that have been thoroughly investigated.^7^

Lattices have been used in a wide range of industrial applications, including aerospace, automotive, construction, and biomedical engineering due to their exceptional properties.^7−9^ These structures can be designed and optimized for specific properties, such as stiffness, strength, energy absorption, and thermal insulation, making them versatile materials for various applications.^10−16^ Architected lattice materials, which are produced by mimicking the crystal microstructure of metals and alloys on the macroscale, are promising candidates for industrial applications.^17−19^ They are made of periodic configurations of nodes and struts usable as macroscopic mechanical mimics of bonds and atoms in crystal structures.^1,2^

The properties of a lattice material are governed by both the distribution of voids and solids as well as the solid ingredient.^20,21^ Various ultralight materials have been created in recent years using a variety of techniques such as polymer foams, metallic foams, ultralight nickel microlattices, and aerogels.^4,12,22−27^ As their density rises, ordered cellular lattice materials become more rigid and durable.^12^

Metallic microlattices have a wide range of potential uses in thermal insulation, battery electrodes, catalyst supports, and acoustic, vibration, or shock energy damping.^15,16,22^ They possess desirable characteristics such as low density, high specific strength, resilience, and absorbance.^4,12,22,28−30^ Furthermore, metallic lattice structures can be fabricated using various manufacturing techniques, directly from the metal material via investment casting, deformation forming, woven and nonwoven metal textiles, and powder bed fusion.^31^ In addition, they can be fabricated from additive manufactured polymer templates that are metallized via electro- or electroless deposition and subsequently removed to achieve the final metallic structure.^4^

To produce metal lattices, normally, nonporous polymer templates are used. Recently, we developed a polymer resin that inherently porous via using emulsion templating and we reported its use as a resin for vat photopolymerization.^32,33^ In particular, polymerized high internal phase emulsions (polyHIPEs), porous polymers produced by a straightforward emulsion templating process, were used.^34^ In this process, two immiscible liquids (in our case a hydrophobic methacrylate phase and water) are mixed stabilized by surfactants or colloidal particles to form a continuous (external) phase and internal (droplet) phase. If the internal phase is more than 74% of the total volume, then the emulsion is classified as a high internal phase emulsion (HIPE).^34−37^ PolyHIPEs are made from HIPEs by further polymerizing the external phase and removing the internal phase.^34,38^ Surfactant-stabilized polyHIPEs typically yield high-surface-area materials with highly interconnected pores, with adjustable and well-defined porosities. In addition to being employed as polymer-based tissue engineering scaffolds, catalytic supports, or filters, polyHIPEs can also be used as templates to create porous metals, ceramics, carbons, and composites.^34,39−45^

Traditional manufacturing methods of polyHIPEs such as casting (molding) can only produce geometrically simple designs and do not offer flexibility in the production of the polyHIPEs.^46,47^ On the other hand, inherently porous and intricate lightweight structures including lattice structures can be fabricated via the combination of emulsion templating with additive manufacturing within a single step.^32,33,48,49^ Among additive manufacturing (AM) techniques, stereolithography (SLA-vat photopolymerization) has the advantage of fabricating the 3D intricate structures directly from the design file with minimum effort. In this AM technique, commercial photocurable resins or emulsion-based resins are used as 3D printing materials.^32,33,48−53^

Via this route, polyHIPEs can be used as templates for producing metallic lattice structures using electroless nickel plating, which is a cost-effective and simple technique for fabricating complex shapes with uniform wall thickness.^4,15,18,28^ Electroless nickel plating is a widely used process for depositing a nickel-phosphorus alloy coating (2–14% phosphorus content) onto a substrate without the need for an external electrical power source.^54,55^ This plating technique involves a chemical reaction between the substrate and plating solution, which results in a uniform coating with precise thickness. Electroless plating can be particularly useful in the production of metallic microlattices, where the precise control of wall thickness is critical to their mechanical properties and functionality.

To deposit a metal coating onto a polymer surface using electroless plating, metal nanoparticle catalysts like Pd, Ag, or Au need to be adsorbed on the surface initially. This process activates the metal cations in the plating solution, which reduce into metal atoms and deposition onto the activated surface. Polymer surfaces are usually inert, however, so pretreatment is needed to introduce functional groups and enhance adhesion between the surface and catalyst. This improves the affinity between the catalyst and surface, promoting uniform and adherent metal deposition during electroless plating.^56^

In this study, the use of additively manufactured polyHIPEs strut structures as templates for electroless nickel plating and their further heat treatment in different atmospheres such as air, argon, and reducing atmospheres was investigated for the first time. The heat treatment in air is intended to remove the polyHIPE polymer substrate, while the argon and reducing atmospheres aim to carbonize the polyHIPE. The first step involved 3D printing polyHIPE discs using a commercial stereolithography-based 3D printer (Elegoo Mars 3 Pro) and optimizing the electroless nickel plating process by varying the coating time. Then, inherently porous lattice structures were 3D printed from high internal phase emulsions and nickel-coated. The effects of various atmospheres during the heating process on the final nickel lattice structure were explored.

## 2 Materials and Methods

### 2.1 Materials

2-Ethylhexyl acrylate (EHA), isobornyl acrylate (IBOA), trimethylolpropane triacrylate (TMPTA), a photoinitiator; diphenyl (2,4,6-trimethyl benzoyl)-phosphine oxide/2-hydroxy-2-methyl propiophenone (blend), beta carotene (synthetic, ≥93% (UV), powder), tartrazine (dye content ≥85%), 3-(trimethoxysilyl)propyl methacrylate, tin (II) chloride (SnCl_2_), palladium (II) chloride (PdCl_2_), boric acid (H_3_BO_3_), and ∼37% hydrochloric acid (HCl) were all purchased from Sigma-Aldrich. The surfactant Hypermer B246-SO-M was donated by Croda. Electroless nickel plating solutions (Part A and Part B) were purchased from Caswell UK.

### 2.2 Methods

#### 2.2.1 Preparation of High Internal Phase Emulsions

39.70 wt % 2-Ethylhexyl acrylate (EHA), 39.70 wt % isobornyl acrylate (IBOA), 15.90 wt % trimethylolpropane triacrylate (TMPTA) (cross-linker), and 4.70 wt % Hypermer B246-SO-M (surfactant) were mixed to form the continuous organic phase (Table 1). The surfactant was dissolved in the mixture by heating it until it was completely dissolved at 50 °C. β-Carotene and tartrazine that were optimized in our previous work were added at 0.02 and 0.06 wt % with respect to the continuous organic phase, respectively, to act as light absorbers.^32^ The photoinitiator was then added to the continuous phase at 5 wt %. Finally, 80 vol % distilled water, dH_2_O, as the internal phase was added dropwise while stirring the mixture at 300 rpm to form an emulsion using a SciQuip-Pro 40 stirrer.

**Table 1:** EHA, IBOA, TMPTA, Hypermer, β-Carotene, and Tartrazine Ratio (wt %)

organic phase				light absorbers	
EHA (wt %)	IBOA (wt %)	TMPTA (wt %)	Hypermer (wt %)	β-carotene^a^ (wt %)	tartrazine^a^ (wt %)
39.70	39.70	15.90	4.70	0.02	0.06

a β-Carotene and tartrazine concentrations with respect to the organic phase.

#### 2.2.2 3D Printing of PolyHIPE Discs and Lattice Structures

PolyHIPE structures were 3D printed using a stereolithography-based commercial 3D printer (Elegoo Mars 3pro). This 3D printer uses a 4K monochrome LCD screen with a resolution of 4098 × 2560 pixels and an XY resolution of 35 μm. The printer also features a chip-on-board lens with integrated UV LED lights paired with a Fresnel lens to deliver an even beam of 405 nm as a light source.^57^

**Figure 1:**
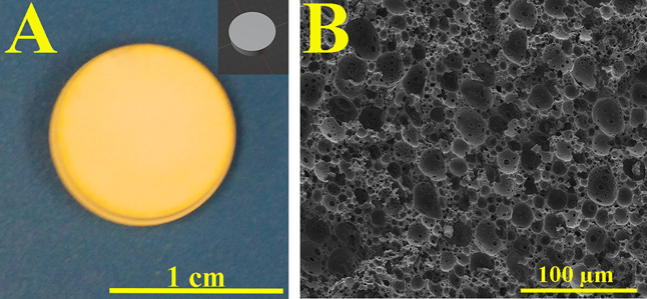
3D-printed polyHIPE disc and the 3D model of the disc (A) and porous internal structure of the 3D-printed polyHIPE (B).

Computer-aided design (CAD) was used to prepare a 10 mm diameter × 2.5 mm height disc (SolidWorks 2018) (Figure 1A). The cubic vertex centroid lattice structure (to be mentioned as “lattice structure” or “lattice” in the text) was obtained from thingiverse.com.^58^ Autodesk Fusion 360 was used to add a base to the lattice structure (Figure 5A). Both designs were formatted as .stl files and then sliced using CHITUBOX Basic.

10 mm diameter × 2.5 mm height discs and 18 mm × 18 mm × 20 mm lattice structures including 20 mm × 20 mm × 3 mm of the base were 3D printed with a layer thickness of 30 μm (Figures 1A and 5B,C). The internal pore size of the 3D-printed polyHIPEs was 14.36 ± 5.78 μm (Figure 1B). The 3D printing parameters including exposure time, bottom layer count, and bottom exposure time etc. are presented in Table 2. To ensure a proper attachment of the structures to the printing platform, the bottom layers should have a higher exposure time than the general exposure time. The exposure time was optimized for each design to prevent overcuring. The overall 3D printing duration was ∼20 min for 30 discs, whereas 18 lattices were 3D printed in ∼3 h in one batch.

**Table 2:** 3D Printing Parameters Used to Produce Discs and Lattices

		exposure time (sec)				
layer height (μm)	bottom layer count	disc	lattice	bottom exposure time (sec)	transition layer count	bottom lift distance/lifting distance (mm)
30	5	10	8	40	5	5

rest time before/after lift (sec)	bottom lift speed (mm/min)	lifting speed (mm/min)	bottom retract speed (mm/min)	retract speed (mm/min)
50	60	70	0	0.5

After 3D printing was completed, 3D-printed structures were washed with methanol to remove any materials eluting from the polyHIPE (e.g., uncured resin) and then dried in an oven at 65 °C for 24 h.

#### 2.2.3 Metallization of 3D-Printed PolyHIPE Discs and Lattice Structures

**Figure 2:**
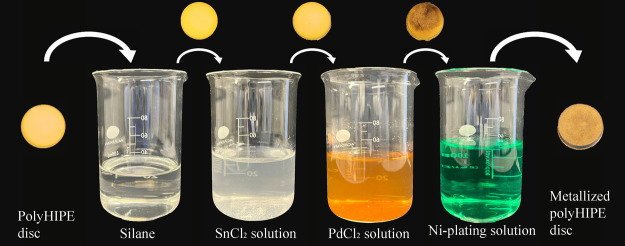
Schematic diagram of the electroless nickel plating process.

The nickel-plating protocol including pretreatments that was published by Sun et al. was used by optimizing it for polyHIPEs.^56^ All steps as a schematic diagram are illustrated in Figure 2.

Before the metallization process, surface functionalization is needed to make the polyHIPE hydrophilic and surface activation is required to enable the deposition of nickel to the substrate.

First, polyHIPEs were immersed in 3-(trimethoxysilyl)propyl methacrylate silane (TMPSM) at 40 °C for 15 min to enhance their hydrophilicity and then dried in an oven at 65 °C until they were fully dried (∼72 h for discs and ∼48 h for lattices). Second, the silane-treated polyHIPEs were immersed in SnCl_2_ and PdCl_2_ solutions at 40 °C for 20 and 10 min, respectively. SnCl_2_ solution was prepared mixing 0.8 wt % SnCl_2_, 100 mL of dH_2_O, and 5 drops of HCl. To prepare 100 mL of PdCl_2_ solution, 0.06 wt % PdCl_2_, 2 wt % H_3_BO_3_, 100 mL of dH_2_O, and 8 drops of HCl were mixed. The pHs of the SnCl_2_ and PdCl_2_ solutions were 1.2 and 1.7, respectively. SnCl_2_ and PdCl_2_ solutions (100 mL) were used to treat 6 polyHIPE discs and 3 polyHIPE lattices to prevent any contamination.

Finally, an electroless nickel plating solution was prepared using Caswell solutions at the following concentrations, 5 vol % Part A, 15 vol % Part B, and 80 vol % dH_2_O.^59^ Surface-activated polyHIPE discs were then immersed in the electroless nickel plating solution at 90 °C for 5 min, 15 min, 30 min, and 1 h. For the polyHIPE lattices, 30 min plating time was applied. The concentrations used to prepare the solutions are presented in Table 3.

**Table 3:** Concentrations of Pretreatment and Electroless Nickel Plating Solutions

silane	SnCl_2_ solution			PdCl_2_ solution				electroless nickel plating solution (Caswell)		
								part		
TMPSM	SnCl_2_	HCl	dH_2_O	PdCl_2_	H_3_BO_3_	HCl	dH_2_O	A	B	dH_2_O
100%	0.8 wt %	5 drops	100 mL	0.06 wt %	2 wt %	8 drops	100 mL	5 vol %	15 vol %	80 vol %

#### 2.2.4 Incineration or Carbonization of the PolyHIPE Template

The polyHIPE templates used to produce metallized polyHIPE lattice structures were incinerated or carbonized in a high-temperature oven (Elite tube furnace, Elite Furnaces, UK). Different atmospheres, such as air, argon (Ar), and reducing (5% H_2_/N_2_), were used to study the incineration or carbonization mechanism of polyHIPEs under various conditions. The temperature was increased to 700 °C with a heating rate of 10 °C/min. After reaching 700 °C, the materials were held for a dwell time of 1 h.

#### 2.2.5 Characterization

##### 2.2.5.1 Mercury Intrusion Porosimetry

The porosity of the polyHIPE discs was measured using a mercury intrusion porosimeter (AutoPore V, Micrometrics) before the metallization process. The highest applied pressure and contact angle of mercury were 60,000 psi (414 MPa) and 130°, respectively.

##### 2.2.5.2 Attenuated Total Reflectance-Fourier Transform Infrared (ATR-FTIR) Spectroscopy

PolyHIPE discs were cut into 300 μm thickness pieces for the ATR analysis once they were silane-treated (PerkinElmer FT-IR Spectrometer Frontier). ATR measurements were obtained from 550 to 4000 cm^–1^ wavenumber at transmittance mode with a resolution of 4 cm^–1^ and 32 scans per sample.

##### 2.2.5.3 X-Ray Diffraction (XRD)

X-Ray diffraction was used to analyze the crystallographic and phase structure of the metallized polyHIPEs discs and lattice structures. XRD was carried out using a PANalytical Aeris, operating at the reduced fluorescence measurement method (Cu tube 30 kV 40 mA, 1/4° divergence slit, a 0.15 mm Ni filter, 0.02 Rad soller slits). The data were collected at diffraction angles (2θs) from 0° to 100° with a step size of 0.02°.

##### 2.2.5.4 Scanning Electron Microscopy/Energy-Dispersive X-Ray Analysis (SEM/EDX)

A FEI Inspect F SEM was used to investigate the internal structure of the nonmetallized and metallized polyHIPEs and the surface of the metallized polyHIPEs. To increase the conductivity of the internal structure of the polyHIPEs, samples were gold-coated before imaging. An accelerating voltage of 5 kV was used for imaging. Pore sizes and nickel thicknesses were measured using ImageJ. Additionally, a statistical correction factor (2/√3) was applied to the pore size measurements to compensate for the underestimation of the diameter caused by uneven sectioning.^60^

Elemental analysis of the Sn- and Pd-treated and metallized samples was done using SEM (Inspect F, FEI) with an energy-dispersive analyzer with 20 kV power.

##### 2.2.5.5 Thermogravimetric Analysis (TGA)

The thermal behavior of the nonmetallized and metallized polyHIPEs (∼5 mg and ∼12 mg, respectively) was determined using thermogravimetric analysis (TGA, Pyris 1, PerkinElmer). Samples were heated in a nitrogen atmosphere from 30 to 1000 °C at a heating rate of 20 °C/min. The weight loss % and increasing temperature relationship of the samples were recorded to identify the thermal characteristic of the polyHIPE samples.

##### 2.2.5.6 Mechanical Testing

Compression testing was performed to evaluate the mechanical properties of the metallized polyHIPE discs (10 mm diameter × 2.5 mm height). A Mecmesin Multitest 2.5 dV mechanical testing machine equipped with a 100 kN load cell was used at a rate of 1 mm/min. The data were obtained using Vector Pro software. The stiffness was determined from the initial linear slope of the stress–strain plot (*n* = 6).

## 3 Results and Discussion

### 3.1 Pretreatment of PolyHIPEs

**Figure 3:**
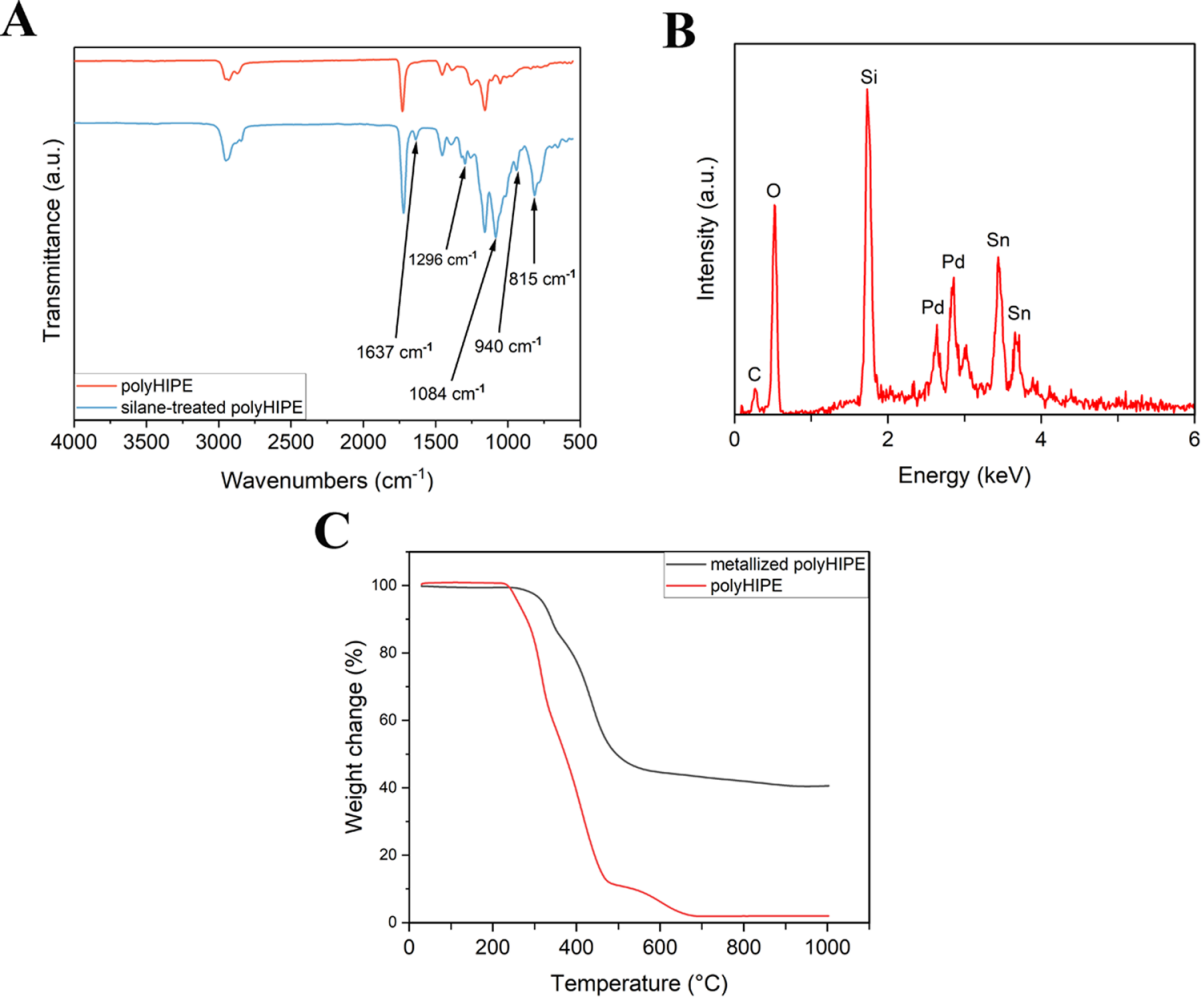
FTIR/ATR graph of the silane-treated polyHIPEs (A), EDX analysis of the Sn- and Pd-treated polyHIPEs (B), and TGA analysis of the nonmetallized and metallized polyHIPEs (C).

FTIR/ATR spectra of the polyHIPEs before and after the silane treatment are presented in Figure 3A. Five new peaks were observed to appear at 815, 940, 1084, 1296, and 1637 cm^–1^. The peak at 815 cm^–1^ was allocated to the bending vibration of C-H, while the peak at 940 cm^–1^ was designated to the stretching vibration of Si-O(H). Additionally, the peaks at 1084 and 1296 cm^–1^ were attributed to the stretching vibration of C-O, and the peak at 1637 cm^–1^ was ascribed to the stretching vibration of C=C.^61,62^ The occurrence of the new peaks indicated that 3-(trimethoxysilyl)propyl methacrylate silane was attached successfully on the polyHIPE surface.

Figure 3B shows the EDX analysis of the polyHIPE samples that were treated with silane, SnCl_2_, and PdCl_2_. The presence of C and O peaks was attributed to the polyHIPE. The successful attachment of the silane was confirmed by the appearance of the Si peak. Moreover, the detection of Sn and Pd peaks in the EDX spectrum indicated that SnCl_2_ and PdCl_2_ were effectively attached to the surface of polyHIPEs.

The TGA analysis of both the nonmetallized and metallized polyHIPEs is shown in Figure 3C. The analysis indicated that the degradation of polyHIPE was completed at 500 °C, and the decomposition occurred between 500 and 700 °C. The residual ash content was approximately 2%. Overall, the TGA graph showed that the polyHIPE was completely incinerated at 700 °C, which is significantly lower than the melting point of the nickel (1453 °C).^12^ Due to this reason, the metallized lattice structures were heated up to 700 °C to remove the polyHIPE template.

### 3.2 Metallization of PolyHIPE Discs

**Figure 4:**
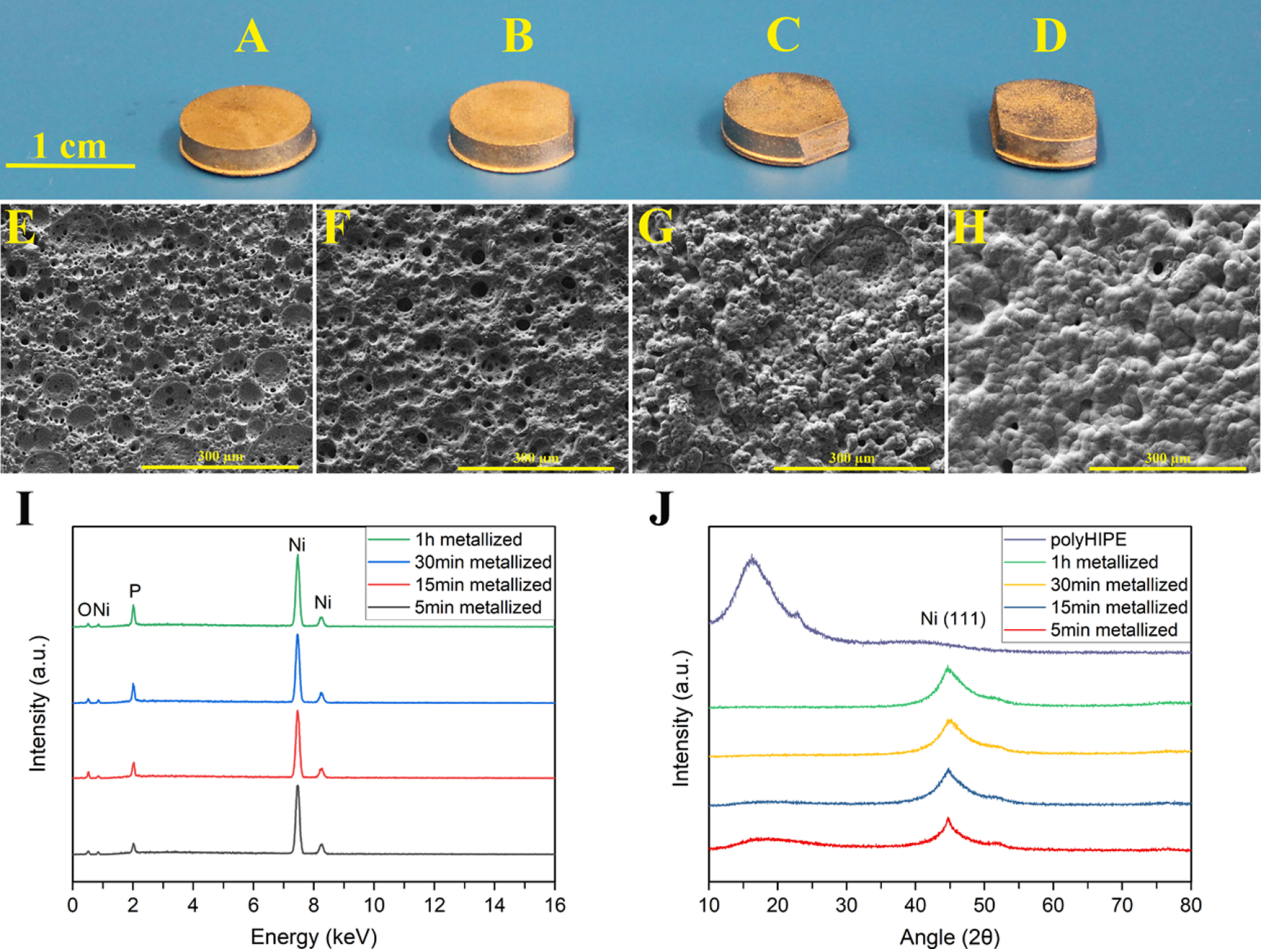
Metallized polyHIPE discs and their SEM micrographs, 5 min (A and E), 15 min (B and F), 30 min (C and G), and 1 h (D and H). EDX (I) and XRD (J) analyses of the metallized polyHIPE discs.

Digital images of polyHIPE discs that were metallized for 5 min, 15 min, 30 min, and 1 h are shown in Figure 4A–D, respectively, and their corresponding SEM micrographs of the bottom of each sample are presented in Figure 4E–H. As expected, they exhibited a metallic appearance. Even though a 5 min metallized sample (Figure 4E) appeared porous, however, at 1 h, the sample had no visible porosity (Figure 4H).

The EDX analysis presented in Figure 4I, along with the XRD analysis shown in Figure 4J, provided further evidence of successful nickel plating. The presence of peaks for nickel (Ni), phosphorus (P), and oxygen (O) was observed in all the sample categories during EDX analysis. The Ni and P peaks were indicative of successful nickel coating, while the O peak could be due to the possible oxidation of the metallized samples.

XRD analysis reveals that Ni (111) peaks were present at 2θ = 44.5° in all the metallized samples.^56^ However, a small broad feature attributed to the polyHIPE was only detected in the 5 min metallized sample at 2θ = 17°. These findings suggest that full coating on polyHIPEs was achieved as the metallization duration increased. The crystallite size as calculated by the Scherrer equation and the degree of crystallinity of the 5 min, 15 min, 30 min, and 1 h metallized samples were 41.3, 20.7, 41.2, and 11.7 nm and 49.66, 78.17, 84.53 and, 89.80%, respectively.

**Figure 5:**
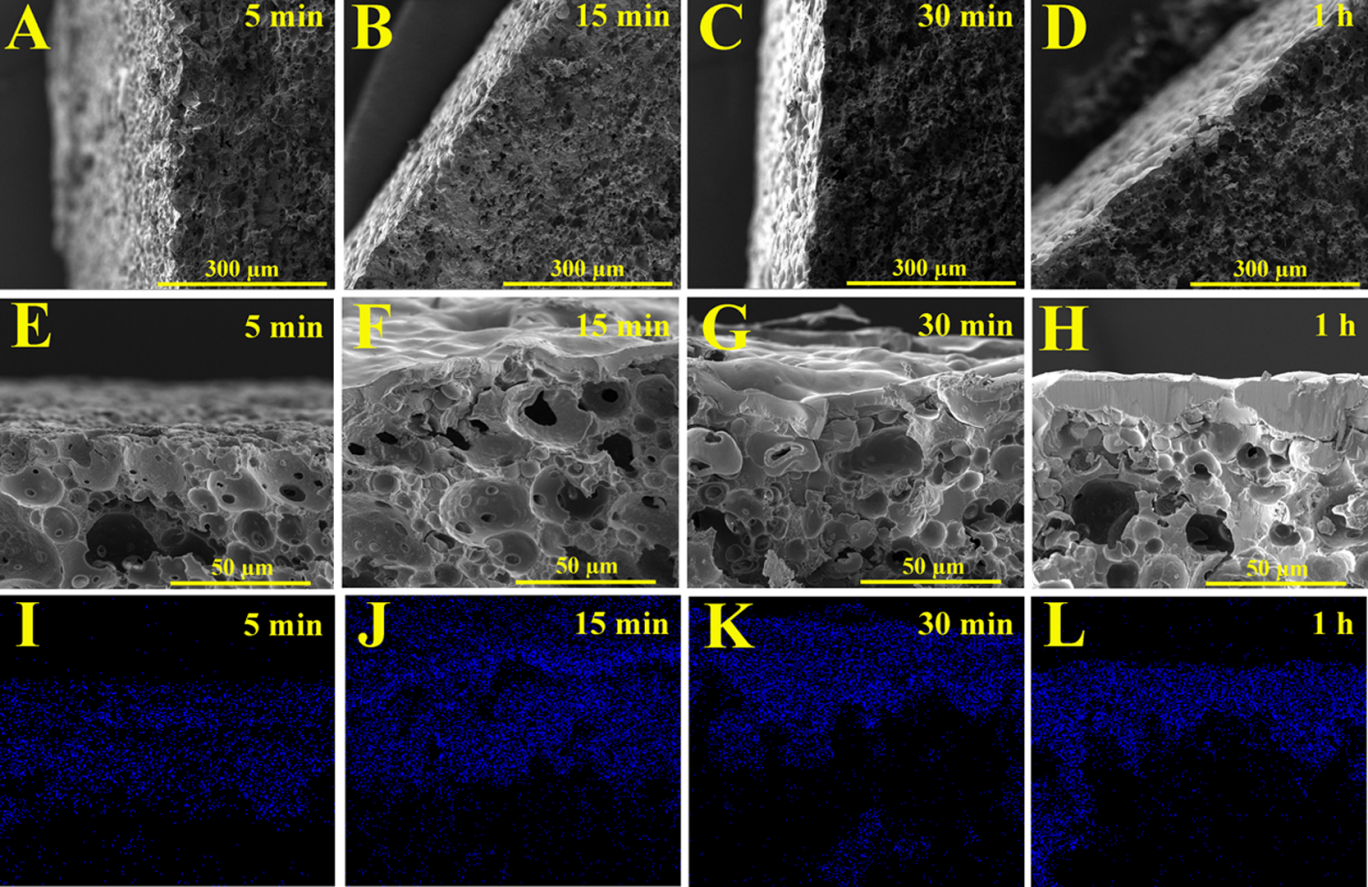
Cross-section SEM images of the metallized polyHIPE discs, 5 min (A), 15 min (B), 30 min (C), and 1 h (D) in lower magnification. Cross-section SEM images of the metallized polyHIPE discs and their corresponding EDX analysis, 5 min (E, I), 15 min (F, J), 30 min (G, K), and 1 h (H, L) in higher magnification.

Furthermore, cross-section SEM micrographs of metallized samples for various time durations (5 min, 15 min, 30 min, and 1 h) are presented in Figure 5A–D. These micrographs showed the nickel layer on the surfaces of the samples. However, as the nickel layer was not thick enough to be seen, higher magnification SEM images of the samples for each time duration, along with corresponding EDX mapping analysis that exhibited the presence of Ni, are illustrated in Figure 5E–H,I–L.

The thickness of a nickel layer deposited on polyHIPEs increases with deposition time between 1.66 ± 0.54 μm for 5 min and 10.20 ± 0.98 μm for 1 h deposition (Table 4). The overall porosity of the nickel-coated samples was around 50%, while the nonmetallized polyHIPE had a higher overall porosity of 74.96%. This indicates that the nickel deposition process does deposit a thin film on the surface of the macrostructure, instead of conformally coating the internal structure of the polyHIPEs. Indeed, this is mainly confirmed by the SEM cross-sections and EDX mapping presented in Figure 5 and indicates that the growing nickel coating reduces the surface porosity of the polyHIPEs and increasingly acts as a barrier for fluid or gas flow in the internal structure by sealing off the surface pores. The results of the EDX mapping analysis shows that still some nickel diffusion occurred into the internal structure because of the surface porosity (Figure 5I–L). This finding supports the idea that, when pores are sufficiently large and interconnected, metal diffusion can occur, resulting in fully metallized pores. Previous literature indicated the possibility of metallizing the internal structure of polyHIPEs, and the EDX mapping results of this study provided further evidence to support this idea.^63,64^

**Table 4:** Porosity (%), Ni Layer Thickness (μm), Weight Change (%), Density (g/cm^3^), and Stiffness (MPa) Values of the Nonmetallized and Metallized polyHIPE Discs

samples	porosity (%)	Ni thickness (μm)	weight change (%)	density (g/cm^3^)	stiffness (MPa)
polyHIPE	74.96	n/a	n/a	1.08	1.12 ± 0.31
5 min metallized	52.77	1.66 ± 0.54	9.88 ± 7.09	1.18	5.04 ± 2.11
15 min metallized	47.72	3.20 ± 0.54	19.44 ± 9.33	1.20	4.60 ± 1.20
30 min metallized	51.05	4.20 ± 1.24	28.32 ± 3.94	1.35	4.20 ± 1.44
1 h metallized	49.63	10.20 ± 0.98	37.76 ± 8.20	1.47	3.87 ± 0.75

In addition to these, the weight change (%) of the metallized samples increased in between 9.88 ± 7.09% for 5 min deposition to 37.76 ± 8.20% for 1 h deposition and this increase leads to an overall increase in density. Table 4 presents the density and stiffness under compression of the printed disks with the increasing nickel-plating time. As expected, the density of the metallized samples increased with the plating time. The nonmetallized polyHIPE had a density of 1.08 g/cm^3^, while the metallized samples had densities ranging from 1.18 g/cm^3^ for 5 min to 1.47 g/cm^3^ for 1 h deposition.

The nonmetallized polyHIPE disc had stiffness of 1.12 ± 0.31 MPa, whereas the stiffness of the 5 min metallized sample increased to 5.04 ± 2.11 MPa. After this initial increase, the stiffness of the disks did not change significantly with thicker nickel coating (from 4.60 ± 1.20 MPa for 15 min to 3.87 ± 0.75 MPa for 1 h coating). This indicates strongly that only the outer surface of the polyHIPE disc is coated (as a nickel shell to the polyHIPE disc) and there is very limited coating of the internal polyHIPE structure.

### 3.3 Metallization of PolyHIPE Lattice Structures

**Figure 6:**
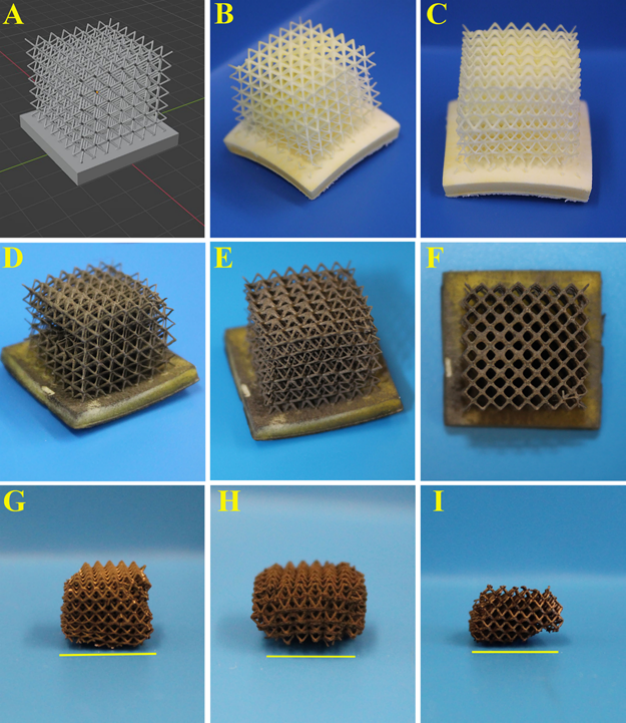
3D design of the lattice structure (A), 3D-printed lattice structure from different perspectives (B, C), nickel-plated lattice structure from different perspectives (D–F), and the lattice structure after the removal of the polyHIPE template in the air (G), argon (H), and reducing (I) atmospheres. (Lattice size in panels B–F is 18 × 18 × 20 mm, and scale bars in panels G–I are 10 mm).

Figure 6A–C illustrates the 3D design of the lattice structure and 3D-printed lattice structure from different perspectives, respectively. The lattice structure had a void size of 1.34 ± 0.26 mm and a strut thickness of 0.27 ± 0.05 mm. It was observed that the 3D-printed design and structure were similar to each other in terms of printing resolution. Figure 6D–F shows the metallized lattice structure from different perspectives, revealing a uniform nickel plating without any deformation in the structure. This highlights that electroless nickel plating provides a uniform coating on intricate structures.

The lattice structures were heated in air, argon, and reducing atmospheres to either remove the polyHIPE template via incineration (in air) or to carbonize the polyHIPE (in argon or reducing atmospheres) after metallization, and the results are presented in Figure 6G–I. As expected, some shrinkage occurred in the lattices after the heating of the polyHIPE template. It was observed that different atmospheres caused similar shrinkages. Specifically, the air, argon, and reducing atmospheres caused 86.11 ± 1.17, 85.81 ± 4.10, and 86.15 ± 4.15 vol % shrinkages in the structure, respectively. The lattice structures heated in the air had more consistent shrinkage than those heated in argon and reducing atmospheres, as indicated by their standard deviations in shrinkage.

**Figure 7:**
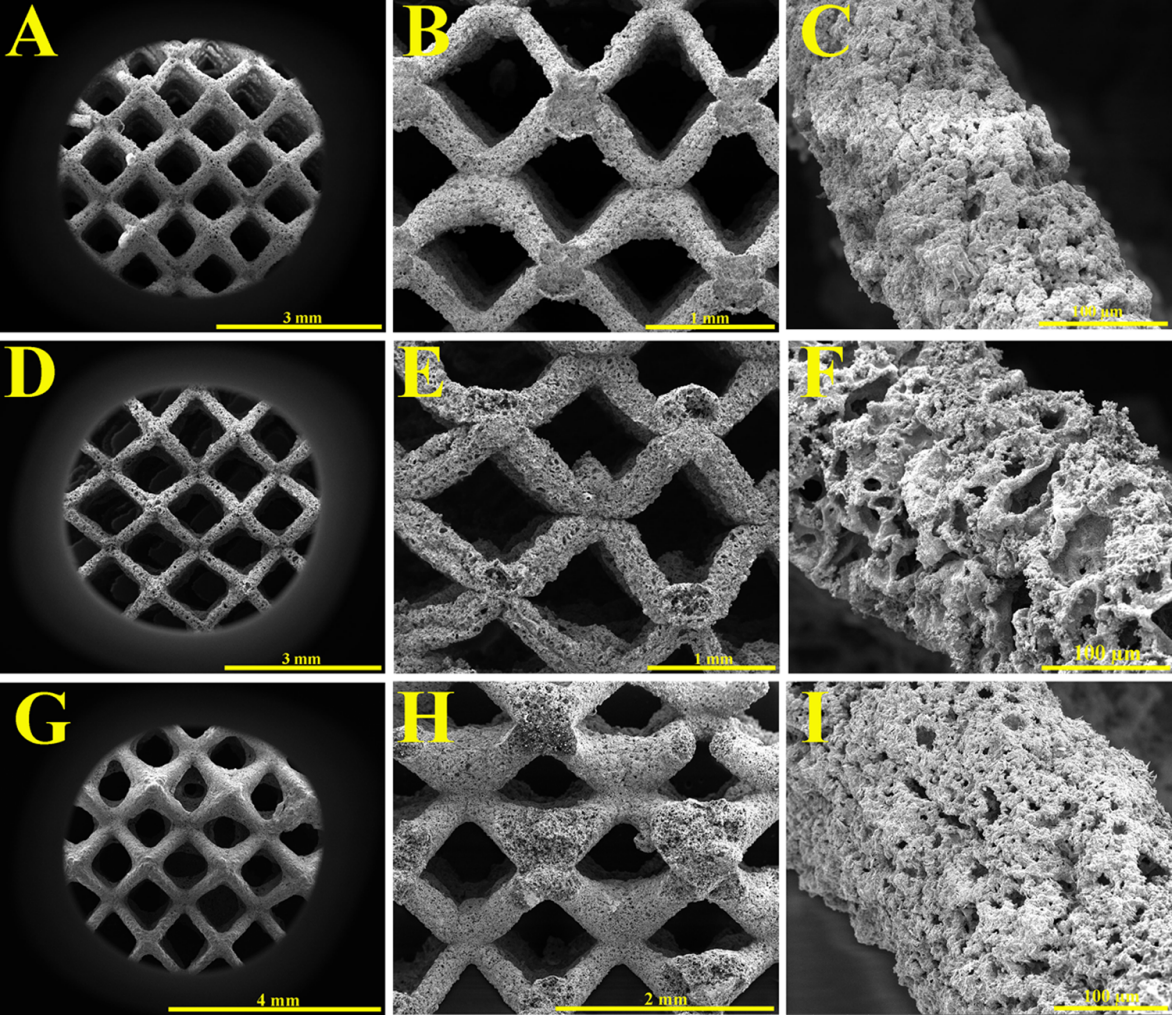
SEM micrographs from the top (A, D, and G), the cross-section (B, E, and H), and the strut in higher magnification (C, F, and I) of the lattice structures heated in air, argon, and reducing atmospheres, respectively.

SEM micrographs of the lattice structures heated in air, argon, and reducing atmospheres are presented in Figure 7, respectively. Figure 7A,D,G shows images taken from the top of the lattice structures, while the cross-section images are presented in Figure 7B,E,H. Finally, one representative strut for each category at higher magnification is exhibited in Figure 7C,F,I. The average strut thicknesses after heating were 0.16 ± 0.03, 0.22 ± 0.05, and 0.19 ± 0.04 mm for the air, argon, and reducing atmospheres, respectively. On the other hand, the strut thickness of the metallized lattice structure was 0.29 ± 0.08 mm. After the removal of the polyHIPE template, the struts of the lattices shrank by an average of 44.83, 24.14, and 34.48% in air, argon, and reducing atmospheres, respectively.

The results indicated that the choice of an atmosphere during the heating process had a significant effect on the shrinkage of the metallic polyHIPE lattice structures. Incineration in air led to a complete combustion and consistent shrinkage in both the macrostructure and struts due to the presence of oxygen, leading to more uniform and consistent shrinkage. In contrast, carbonization in argon or reducing atmospheres led to less shrinkage in the struts but significant shrinkage in the macrostructure. However, in inert or reducing atmospheres, there was no combustion, and we observed still an internal porous carbon scaffold within the struts. In an air atmosphere, the nickel struts are completely empty. Interestingly, the macrostructure still shrank isotropically, without destroying the 3D strut structure. Also, it is interesting to observe from the SEM figures (Figure 7C,F,I) is that all three nickel coatings are highly porous.

**Figure 8:**
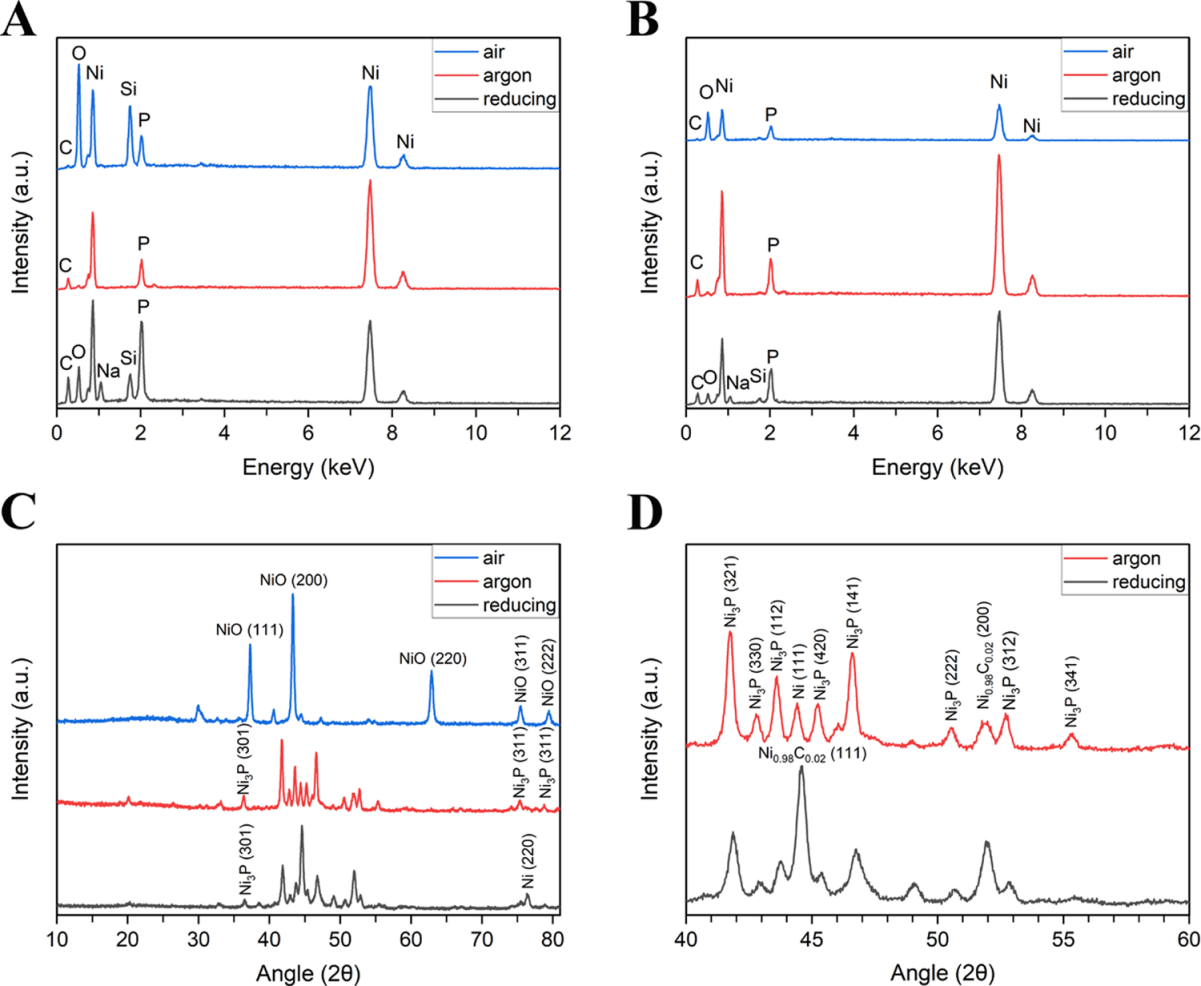
EDX analysis of the lattice surfaces (A) and EDX analysis of the strut from the cross-section of the lattices (B). XRD analysis of the lattices (C) and the zoomed-in XRD analysis graph (D).

Figure 8 displays the EDX and XRD analyses of the lattice structures after heating. Figure 8A,B shows the EDX analysis performed on the surface of the lattice structures and struts, respectively. The presence of nickel (Ni) peaks in all three conditions confirmed the effective nickel plating on both the lattice surface and struts. These results demonstrated the feasibility of uniformly coating intricate polyHIPE structures using the electroless plating technique. Moreover, the formation of phosphorus (P) and sodium (Na) peaks could be attributed to the electroless nickel plating solution, whereas the silicon (Si) peak was observed due to the application of silane to enhance the hydrophilicity of the polyHIPEs.

The XRD analysis of the lattices and the zoomed-in XRD analysis to observe the peaks clearly are presented in Figure 8C,D, respectively. Concerning the XRD analysis, in air-heated lattices, only NiO peaks were present, indicating that the lattice structure was fully oxidized in an air atmosphere (NiO: PDF-4+ ICDD 04-006-6925). Meanwhile, in argon-heated lattices, metallic nickel (Ni), nickel phosphide (Ni_3_P), and nickel carbide (Ni_0.98_C_0.02_) peaks were observed (Ni_3_P: PDF-4+ ICDD 01-089-4748, Ni: PDF-4+ ICDD 04-010-6148, Ni_0.98_C_0.02_: PDF-4+ ICDD 01-074-5561). Furthermore, very similar compounds (Ni and Ni_3_P) were formed in the reducing atmosphere-heated lattices (Ni_3_P: PDF-4+ ICDD 01-089-4748, Ni: PDF-4+ ICDD 04-010-6148, Ni_0.98_C_0.02_: PDF-4+ ICDD 01-074-5561). No carbon related peaks were observed indicating that the carbon phase is highly amorphous. The XRD identifies both metallic nickel and nickel phosphide (Ni_3_P) under inert and reducing atmospheres.

During the electroless nickel plating process, a variety of nickel-phosphorus alloys are deposited on the substrate according to the phosphorus concentration.^55^ The metallurgical characteristics of the alloys are determined by the amount of phosphorus, which can range from 2 to 14%.^55^ The previous work on the nickel coating of polyHIPEs showed the formation of different forms of nickel phosphides (N_12_P_5_, Ni_2_P, and Ni_3_P).^64^ However, the solid-state reaction occurs at temperatures of 600 °C or higher, and Ni_3_P is specifically created when there is an excess of nickel.^65^ Due to the treatment temperature of 700 °C and the higher concentration of nickel relative to phosphorus (Figure 4I) in our experimental conditions, Ni_3_P was anticipated to form. Interestingly, Ni_3_P is known as a high-performance catalytic phase.^66^ The formation of this compound indicates that these structures can be good candidates for the catalytic applications.

The final observation indicated that all three types of lattices became brittle after the heat treatment, with the lattice heated in a reducing atmosphere being the most brittle.

In addition to these, Scherrer analysis was performed on the aforementioned peaks, revealing average crystallite sizes of 27.96 nm (NiO), 31.92 nm (Ni_3_P), and 26.92 nm (Ni_3_P) in air, argon, and reducing atmospheres, respectively.

**Figure 9:**
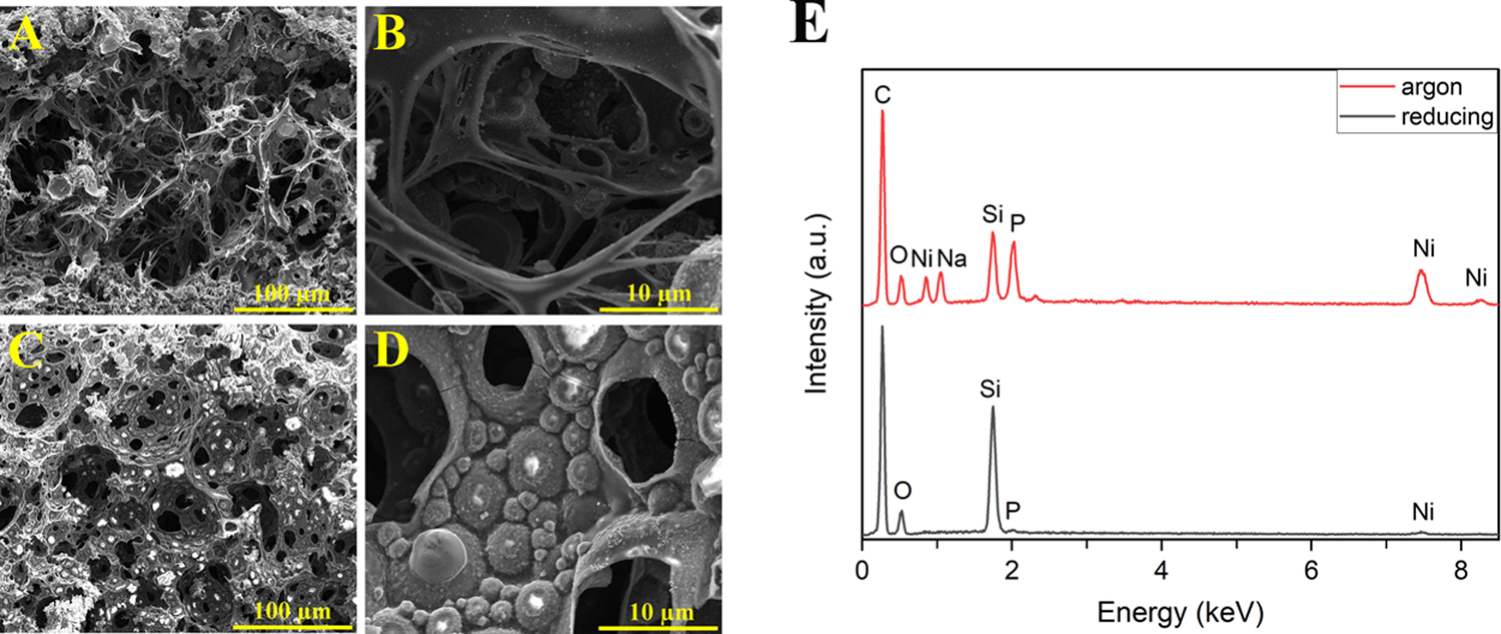
Internal porous structure of the lattice structures burned in argon (A, B) and reducing (C, D) atmospheres. EDX analysis of the internal porous structures (E).

As mentioned, the lattice structures heated in argon and reducing atmospheres have an amorphous carbon network remnant, within the struts as illustrated in Figure 9A,B and Figure 9C,D, respectively. EDX analysis was carried out in high-magnification SEM micrographs (Figure 9B,D). The findings revealed that the carbon (C) peak had the highest intensity, indicating the presence of the internal porous carbon structure in argon and reducing atmospheres. Moreover, the analysis detected the formation of nickel (Ni) and phosphorus (P) peaks, demonstrating the occurrence of some nickel/nickel phosphide nanoparticles in the internal structure.

## 4 Conclusions

Electroless nickel-plating was examined and optimized to create ultra-porous metallized structures on polymerized high internal phase emulsions (polyHIPEs). To achieve this, 3D-printed discs were fabricated using high internal phase emulsions (HIPEs) and metallized for various durations (5 min, 15 min, 30 min, and 1 h) with the electroless nickel-plating technique. The process was investigated using XRD, SEM, and EDX analyses. Subsequently, 3D-printed polyHIPE lattice structures were uniformly coated with nickel. To either remove or carbonize the polyHIPE templates, the metallized lattice structures were heated in air, argon, and reducing atmospheres. The nickel coating remained intact after the polyHIPE templates were removed. Heating polyHIPEs in different atmospheres led to the formation of various compounds. Metallic polyHIPEs heated in an air atmosphere were entirely oxidized, while nickel metal and nickel phosphide (Ni_3_P) hybrid structures formed in argon and reducing atmospheres. Additionally, the internal structure of the polyHIPEs was fully carbonized while preserving the porous structure in argon and reducing atmospheres.
