# Correction to “Investigating the Potential of Electroless Nickel Plating for Fabricating Ultra-Porous Metal-Based Lattice Structures Using PolyHIPE Templates”

**DOI:** 10.1021/acsami.3c16884

**Published:** 2023-12-01

**Authors:** Nihan Sengokmen-Ozsoz, R. Boston, Frederik Claeyssens

In our original paper, we mistakenly put in the wrong printing parameters in Table 2 on page 30771. In the corrected Table 2 below, we present the correct printing parameters. This correction does not alter any of the conclusions of the paper.

**Table 2:** 3D Printing Parameters Used to Produce Discs and Lattices

		Exposure time (sec)				
Layer height (μm)	Bottom layer count	Disc	Lattice	Bottom exposure time (sec)	Transition layer count	Bottom lift distance/Lifting distance (mm)
30	5	10	8	40	5	5

Bottom lift speed(mm/min)	Lifting speed (mm/min)		Bottom retract speed/Retract speed (mm/min)		Rest time before/after lift (sec)	Rest time after retract (sec)
60	70		50		0	0.5

